# Optimized saturation recovery protocols for T1-mapping in the heart: influence of sampling strategies on precision

**DOI:** 10.1186/s12968-014-0055-3

**Published:** 2014-09-04

**Authors:** Peter Kellman, Hui Xue, Kelvin Chow, Bruce S Spottiswoode, Andrew E Arai, Richard B Thompson

**Affiliations:** National Heart, Lung, and Blood Institute, National Institutes of Health, DHHS, 10 Center Drive MSC-1061, Bethesda, MD 20892 USA; Siemens Medical Solutions, Chicago, USA; Department of Biomedical Engineering, University of Alberta, Edmonton, Canada

**Keywords:** T1 mapping, Random error, Precision, SASHA, Saturation recovery, Myocardial fibrosis

## Abstract

**Background:**

T1-mapping has the potential to detect and quantify diffuse processes such as interstitial fibrosis. Detection of disease at an early stage by measurement of subtle changes requires a high degree of reproducibility. Initial implementation of saturation recovery (SR) T1-mapping employed 3-parameter fitting which was highly accurate but was quite sensitive to noise; 2-parameter fitting greatly reduced the sensitivity to noise at the expense of a small degree of systematic bias. A recently introduced implementation that uses a variable readout flip angle greatly reduces systematic errors in T1-measurement thereby making it feasible to use SR methods with 2-parameter fitting with improved accuracy and precision. SR T1 mapping techniques with multi-heartbeat recovery times have been proposed to better sample the T1 recovery curve, but have not been evaluated for 2-parameter fitting.

**Methods:**

An analytic formulation for calculating the standard deviation (SD) for SR T1-mapping with 2-parameter fitting is developed and validated using Monte-Carlo simulation. The coefficient of variation is compared for a brute force optimization of sampling and for several previously described sampling schemes for T1 measurement over several uncertainty ranges. Experimental validation is performed in phantoms over a range of T1, and in-vivo both native and post-contrast. Pixel-wise SD maps are calculated for SR T1-mapping.

**Results:**

Sampling schemes that use a non-saturated anchor image and multiple (N) measurements at a single fixed saturation delay are found to be near optimum for the case of known T1 and are close to the brute force optimized solution over wide ranges of native and post-contrast T1 values. The fixed delay sampling scheme is simple to implement and provides an improvement over uniformly distributed schemes.

**Conclusions:**

Sampling strategies for saturation recovery methods for myocardial T1-mapping have been optimized and validated experimentally. Reduced SD, or improved precision, may be achieved by using fixed saturation delays when considering native myocardium and post-contrast T1 ranges. Pixel-wise estimates of T1 mapping errors have been formulated and validated for SR fitting methods.

## Background

T1-mapping has the potential to detect and quantify diffuse myocardial processes such as interstitial fibrosis. Detection of disease at an early stage by measurement of subtle changes requires a high degree of reproducibility [1]. Reproducibility is fundamentally limited by precision and may be further limited by systematic variations [2,3]. The recently proposed SAturation recovery with single-SHot Acquisition (SASHA) method [4] has been introduced in an effort to reduce systematic measurement biases [2,3] due to factors such as off-resonance and flip angle variation, dependence on T2, heart rate, and sensitivity to protocol parameters compared to the MOdified Look-Locker Inversion recovery (MOLLI) sequence [5]. SASHA has also been shown to be less dependent on magnetization transfer (MT) than MOLLI and its variants [6]. Initial implementation of SASHA T1-mapping employed 3-parameter fitting which was highly accurate but was quite sensitive to noise; 2-parameter fitting greatly reduced the sensitivity to noise at the expense of a small degree of systematic bias [3,4]. The bias error arises due to the influence of the readout pulses following the saturation pulse that precede the center of k-space. A recently introduced implementation of SASHA [7] that uses a variable readout flip angle (VFA) greatly reduces systematic errors in T1-measurement thereby making it feasible to use 2-parameter fitting with improved accuracy and precision. The use of VFA reduces the influence of the readout prior to reaching the center of k-space since the flip angle is reduced during this period. VFA also reduces image artifacts that arise due to oscillations during the transient approach to steady state, particularly the ghosting of fat due to off-resonance.

SASHA was originally described with saturation recovery times (TS) limited to a single heartbeat to allow the maximum number of images to be acquired within a given duration. Longer TS times can be obtained by playing the image readout in the heartbeat following the saturation pulse, as previously described for the SR-TFL sequence [8] and more recently in Saturation Method using Adaptive Recovery times for cardiac T1 Mapping (SMART1Map) [9,10]. These multi-heartbeat TS times have the advantage of sampling a greater portion of the recovery curve, although fewer images are acquired for a fixed total duration. The net effect in precision of calculated T1 values has not been previously explored.

Precision relates to random errors due to noise and is a function of the number and timing of measurements along the T1-saturation recovery curve. We examine the effect of sampling on precision including the position (i.e., SR delay TS) and number of samples, and propose an optimized sampling scheme to reduce the error due to noise. We provide an analytic formulation for the calculation of the standard deviation (SD) for 2-parameter saturation recovery fitting and validate this formulation using Monte-Carlo simulation. Pixel-wise maps of noise SD can be used as a quality or confidence map and may be generated using this formulation in the same manner as for inversion recovery SD maps [11]. A comprehensive analysis and optimization has been described for fitting with 3 parameters [12,13] that leads to different results, however 3 parameter fitting is not considered here since it is significantly more sensitive to noise which greatly reduces precision [3].

We proposed a sampling strategy that uses a non-saturated anchor image and N measurement at a fixed saturation delay (TS). We calculate the optimum fixed TS for a given T1 and number of measurements N, and correspondingly determine the optimum achievable SD. We compare sampling strategies where T1 is in a known range and consider a range for native myocardial T1 mapping, a range for post-contrast, and a wider range spanning T1 values for both native and post-contrast. Sampling strategies that were compared include using a non-saturated anchor image plus a) fixed TS for all measurements, b) uniform distribution of TS over the heart interval [4], c) brute force optimization, and d) strategies employing multiple recovery heartbeats to obtain longer TS samples [10].

Comparisons of protocols with different sampling strategies are validated in phantoms and in-vivo. Experimental measurements are in excellent agreement with numerical prediction based on theory.

## Methods

### Theory

The 2-parameter model for saturation recovery may be written as:

1 $$ y\left(\mathrm{TS}\right)=A\left(1- \exp \left(-\frac{TS}{T1}\right)\right) $$

where *y*(TS) is longitudinal magnetization, T1 is the longitudinal recovery time constant, TS is the saturation recovery time and A is the signal amplitude. The desired covariance matrix C of the estimated parameters (A and T1) may be approximated as [11]:

2 $$ \mathbf{C}=\left[\begin{array}{cc}\hfill {\sigma}_{T1}^2\hfill & \hfill \cdot \hfill \\ {}\hfill \cdot \hfill & \hfill {\sigma}_A^2\hfill \end{array}\right]={\left({\displaystyle \sum_i\frac{1}{\sigma_i^2}}\left[\begin{array}{cc}\hfill \frac{\partial y\left(T{S}_i\right)}{\partial T1}\cdot \frac{\partial y\left(T{S}_i\right)}{\partial T1}\hfill & \hfill \frac{\partial y\left(T{S}_i\right)}{\partial T1}\cdot \frac{\partial y\left(T{S}_i\right)}{\partial A}\hfill \\ {}\hfill \frac{\partial y\left(T{S}_i\right)}{\partial A}\cdot \frac{\partial y\left(T{S}_i\right)}{\partial T1}\hfill & \hfill \frac{\partial y\left(T{S}_i\right)}{\partial A}\cdot \frac{\partial y\left(T{S}_i\right)}{\partial A}\hfill \end{array}\right]\right)}^{-1} $$

where *TS*_*i*_ are the saturation delays for each sample *i*, and σ_i_ = σ is the standard deviation of the signal y which are assumed to be independent and identically distributed; ${\sigma}_{T1}^2$ and ${\sigma}_A^2$ represent the variance of T1 and A, respectively. The partial derivatives in Eq 2 for the 2-parameter signal model Eq 1 are:

3 $$ \frac{\partial y}{\partial A}=1- \exp \left(-\frac{TS}{T1}\right) $$

$$ \frac{\partial y}{\partial T1}=-A\cdot \exp \left(-\frac{TS}{T1}\right)\cdot \frac{TS}{T{1}^2} $$

The desired variance for the parameter T1 may be calculated using the analytic 2×2 inverse of Eq 2.

4 $$ {\sigma}_{T1}^2={\sigma}^2\frac{{\displaystyle \sum \frac{\partial y\left(T{S}_i\right)}{\partial A}\cdot \frac{\partial y\left(T{S}_i\right)}{\partial A}}}{{\displaystyle \sum \frac{\partial y\left(T{S}_i\right)}{\partial T1}\cdot \frac{\partial y\left(T{S}_i\right)}{\partial T1}}{\displaystyle \sum \frac{\partial y\left(T{S}_i\right)}{\partial A}\cdot \frac{\partial y\left(T{S}_i\right)}{\partial A}-{\left({\displaystyle \sum \frac{\partial y\left(T{S}_i\right)}{\partial T1}\cdot \frac{\partial y\left(T{S}_i\right)}{\partial A}}\right)}^2}} $$

After substituting the partial derivatives in Eq 3 into Eq 4, assuming there are N measurements at *TS*_*i*_ plus a non-saturated anchor image at *TS* = ∞, and simplifying, one may derive an expression for the square of the coefficient of variation times SNR, ${\left(\frac{\sigma_{T1}}{T1}\times SNR\right)}^2:$

5 $$ {\left(\frac{\sigma_{T1}}{T1}\times SNR\right)}^2=\frac{1}{\left({\displaystyle \sum_{i=1}^N{\left( \exp \left(-\frac{T{S}_i}{T1}\right)\cdot \frac{T{S}_i}{T1}\right)}^2}\right)-\frac{{\left({\displaystyle \sum_{i=1}^N\left( \exp \left(-\frac{T{S}_i}{T1}\right)\cdot \frac{T{S}_i}{T1}\right)\cdot \left(1- \exp \left(-\frac{T{S}_i}{T1}\right)\right)}\right)}^2}{1+{{\displaystyle \sum_{i=1}^N\left(1- \exp \left(-\frac{T{S}_i}{T1}\right)\right)}}^2}} $$

where SNR = A/σ. Thus the coefficient of variation $\frac{\sigma_{T1}}{T1}$ is a function of T1, SNR, and the saturation delays *TS*_i_ and may be readily evaluated numerically.

### Sampling strategies and notation

**Figure 1:**
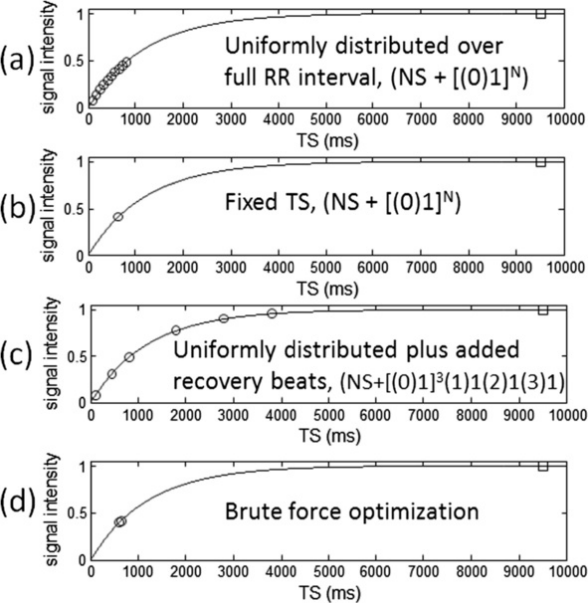
**Saturation recovery sampling strategies considered in protocol comparisons:** **(a) uniformly distributed saturation delays,** **(b) fixed saturation delay,** **(c) uniformly distributed saturation delays plus added recovery beats, and** **(d) brute force optimization of sampling.** All schemes acquired an initial non-saturated “anchor” image (plotted at 9500 ms) and all protocol comparisons had equal duration acquisitions (RR = 1000 ms in this illustration).

In order to compare various sampling strategies for SASHA that includes the possibility for recovery heartbeats to permit saturation delays TS > RR (the duration of a single cardiac cycle) as used in SMART_1_Map [10], a notation is introduced here. All of the protocols considered here acquire an initial non-saturated image referred to as NS as well as additional measurements at various saturation delays. For instance, the SASHA protocol described in the original publication [4] would acquire a total of 10 images consisting of NS plus 9 additional SR images would be written here as NS + [(0)1]^9^ uniform, where the (0) indicates there were no recovery beats between SR image measurements, and the [ ]^9^ indicates 9 measurements distributed uniformly across the RR interval. The SMART_1_Map acquisition scheme described in [8] can be written as NS + [(0)1]^3^(1)1(2)1(3)1 uniform, where there is a NS plus 3 images acquired without recovery with uniformly distributed saturation delays followed by 3 additional images acquired with 1, 2, and 3, recovery beats, respectively, assumed to be acquired at the maximum available saturation delay for the given heart rate. This would correspond to a total acquisition of 7 images in 13 heartbeats including the NS and 6 recovery beats for which there are no images acquired. For comparison of SD of T1-measurements between these different protocols, the total acquisition time was considered fixed. Since the reported SMART_1_Map protocol was 13 heart beats, a 13 heart beat SASHA protocol was used for comparison. Therefore, 13 heartbeat SASHA protocols were considered NS + [(0)1]^12^ with both uniformly distributed TS as in the original SASHA [4] and a modified protocol with fixed TS, i.e., all measurements at the same saturation recovery delay chosen through numerical optimization to be described. These protocols are illustrated in Figure 1(a-c). Multiple measurements at the same saturation delay were not averaged. Fitting was performed on all measurements including the multiple measurements at the same saturation delay, TS.

### Optimization of sampling strategies

The coefficient of variation (CV) was evaluated per Eq 5 for a number of cases. Firstly, it was assumed that T1 was known and the optimum sampling strategy was determined. Alternatively, it was assumed that T1 was known in a finite range and the optimum sampling strategy was determined over ranges corresponding to native contrast, post-contrast, and a wide range which encompassed both native and post contrast. The ranges were chosen broadly to be 1000–1400 ms (native), 250–600 ms (post-contrast), and 250–1400 ms (wide) to encompass a range of conditions. Optimization consisted of choosing a set of saturation delays, TS_i_, and computing the CV across the specified range of T1. A brute force search was conducted by calculating all possible sets of delays TS_i_ selected over a grid from TS min to TS max with replacement, i.e. allowing repeated TS values. The scheme with the minimum worst case CV over the T1 range was selected as optimal. For example, if one were to specify TS min = 100 ms and TS max = 800 ms, with an increment of 50 ms, then there would be N_incr_ = 15 possible values for TS. For a protocol such as NS + [(0)1]^12^ with N_TS_ = 12 values chosen over N_incr_ = 15, the number of possible cases with replacement would be:

6 $$ Ncases=\left(\left(\begin{array}{c}\hfill Nincr\hfill \\ {}\hfill {N}_{TS}\hfill \end{array}\right)\right)=\left(\begin{array}{c}\hfill {N}_{TS}+ Nincr-1\hfill \\ {}\hfill {N}_{TS}\hfill \end{array}\right)=\frac{\left({N}_{TS}+ Nincr-1\right)!}{N_{TS}!\left( Nincr-1\right)!} $$

or in this example, 9,657,700. Calculations were performed using MATLAB (Mathworks, Natick, MA USA) and Eq 5 was vectorized to compute the max(CV) across a range of T1’s in 10 ms increments. In this example, the brute force calculation for the native or post-contrast T1 ranges would test all the cases in approx. 2.5 min, with each case calculated in less than 16 microsec. The brute force optimization was performed at HR = 60 bpm using Nincr = 15 samples between TS min = 100 and TS max = 800, and at HR = 120 bpm using Nincr = 15 samples between TS min = 100 ms and TS max = 350 ms. The brute force optimization was extended to include up to 8 recovery beats. All possible combinations of recovery/acquisition were considered that summed to a total duration of 12 beats. Values of TS for measurements that followed recovery were assumed to be maximized, i.e., equal to Nrecovery*RR + TS max. Brute force optimizations were calculated for HR’s 60 and 120 bpm. The brute force optimization was performed both considering recovery heartbeats and without any recovery heartbeats.

The sampling strategies considered are illustrated in Figure 1 and panel (a) with uniform distribution over the RR corresponds to the original published SASHA scheme [4].

### Numerical validation of SD formulation

A Monte-Carlo simulation using N = 65,536 trials was used to compute the standard deviation (SD) in T1 as a function of SNR and T1 for a specific SASHA protocol (NS + [(0)1]^12^) and was compared with the estimate of standard deviation based on the analytic formulation.

### Imaging

Imaging was performed on a 1.5 T Siemens Aera (Siemens Medical Solutions, Erlangen, Germany), equipped with a 45 mT/m and 200 T/m/s gradient systems. The original uniform SASHA sampling strategy, the fixed TS sampling strategy, and the SMART_1_Map sampling strategy with recovery beats were used. For phantom imaging, the fixed TS sampling strategy had 3 protocols: (a) optimized for native (pre-contrast) myocardial T1 values, (b) optimized for shorter T1 corresponding to Gd contrast, and (c) optimized for the wide range of T1 covering both pre- and post-contast ranges. All protocols had an acquisition of 13 heart beats. All in-vivo imaging was acquired using breath-holding. Non-rigid motion correction was used to correct any residual in-plane respiratory motion.

Imaging parameters for all sampling schemes were: non-selective adiabatic saturation pulse, steady state free precession single shot read out with variable flip angle [7] which smoothly approached a 70° excitation flip angle, typical field of view 360 × 270 mm^2^, slice thickness 8 mm, matrix 256×144, voxel size 1.4 × 1.9 × 8.0 mm^3^, TR/TE 2.7/1.1 ms, 7/8 partial Fourier plus parallel imaging factor 2 using separate reference lines acquired at the completion of the scan. The BIR4-90 saturation pulse was designed to achieve saturation to within 0.6% over an off-resonance range ±125 Hz and between 75 and 100% of design flip angle.

### Phantom measurements

Phantom validation used a set of CuSO4 doped agar gel phantoms with varying concentrations with T1 and T2 in the expected range for myocardium, both native and with Gd contrast. Phantoms had T1 in the range 250–1600 ms and T2 in the range 40–75 ms. The agar tubes were in a bath of saline doped with Gd with T1 approx. 220 ms.

**Figure 2:**
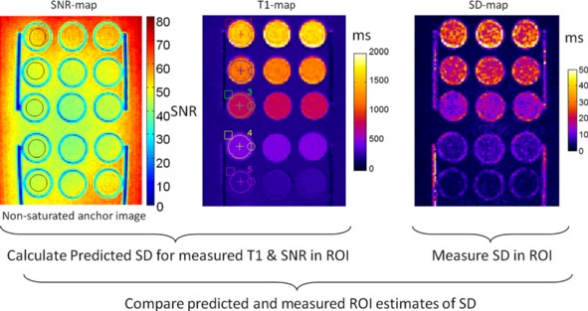
**Methodology used for phantom ROI analysis comparing predicted SD based on measured SNR and T1 with measured SD.**

The T1, SD, and SNR were measured in 5 ROIs with varying T1 and the measured values of T1 and SNR were used for numerical calculation of a predicted SD, which was compared with the measured SD in each ROI. The methodology is illustrated in Figure 2. Images were reconstructed with scale in SNR units [14,15]. SNR maps were calculated from the signal intensities of the non-saturated image.

### In-vivo studies

In-vivo data was acquired using 3 imaging protocols with the same 3 sampling strategies defined above (original SASHA, fixed TS, and SMART_1_Map) to compare the T1 map SD for both native myocardial T1 and following administration of contrast. In-vivo imaging used the native contrast optimized protocol for native contrast imaging (TS = 600 ms) and the post-contrast optimized protocol for post-contrast imaging (TS = 200 ms). Both pre- and post-contrast datasets were acquired in n = 10 subjects. A paired t-test was used to assess the statistical significance between measurements of the SD in a septal ROI. This study was approved by the local Institutional Review Boards of the National Heart, Lung, and Blood Institute and Suburban Hospital, and all subjects gave written informed consent to participate. Post-contrast T1-maps were typically acquired at least 15 min following administration of Gd contrast (0.15 mmol/kg) (Gadavist, Bayer Healthcare).

## Results

### Analytic SD validation by Monte-Carlo simulation

**Figure 3:**
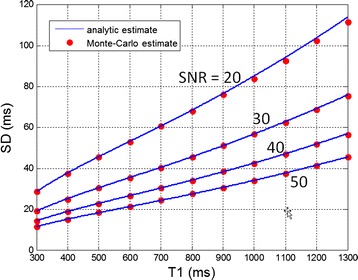
**Validation of analytic formulation of SD using Monte-Carlo with n = 65536 trials demonstrating excellent agreement.**

Using SASHA with protocol NS + [(0)1]^12^ with uniformly distributed TS, the predicted SD was within 1% of measured Monte-Carlo SD for SNR ≥ 30, and within approx. 2% of measured SD for SNR = 20 (Figure 3).

### Optimization for known T1

**Figure 4:**
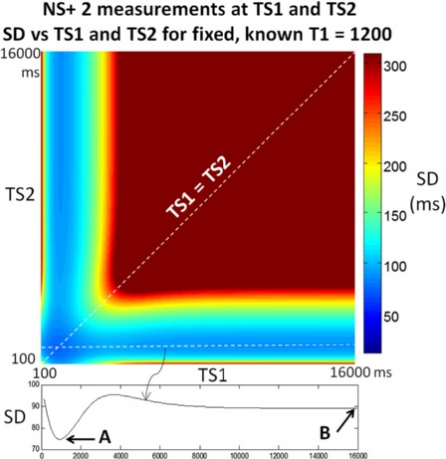
**SD plotted vs TS**_**1**_ **and TS**_**2**_ **for case of NS + 2 SR measurements without constraints on TS.** The global minimum SD for an assumed known T1 = 1200 ms is achieved with a fixed TS = TS_1_ = TS_2_ = 910 ms (arrow A). Note there is a local minimum corresponding to TS_1_ = 1090, with very long TS_2_ (arrow B) which is effectively 1 SR measurement and 2 non-saturated anchor images.

In the case where T1 is assumed to be known, brute force optimization found that the sampling strategy of using all measurements with a single fixed value of TS plus a non-saturated anchor image without added recovery beats was a near optimum distribution. For sufficiently large N, low T1, and low HR, the addition of a single measurement with recovery heart beats to achieve a longer saturation delay was found to reduce the CV. Consider the case of NS + 2 measurements as an illustration. The SD(TS_1_,TS_2_) for a protocol with NS + 2 measurements (NS + [(0)1]^2^) is minimum along a line TS_1_ = TS_2_ (Figure 4). In this illustration, the saturation delays are not-constrained to be less than the RR interval as they would in a real implementation in order to visualize the surface and various minima. There is a global optimum (arrow A) at TS_1_ = TS_2_ < T1 where TS is relatively short and there is another relative minimum at a point (arrow B) with short TS_1_ < T1 and a long TS_2_ > > T1 which would necessitate many recovery heart beats to achieve.

**Figure 5:**
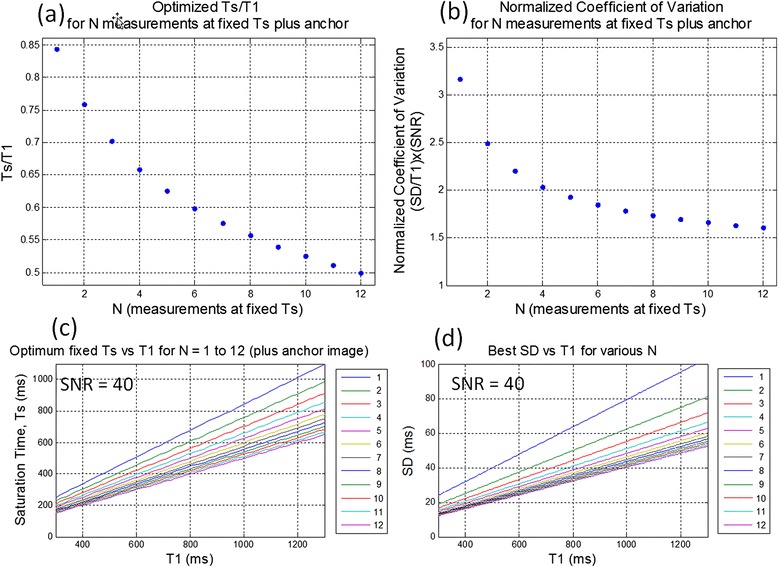
Optimization for fixed TS with N measurements plus NS anchor (a) the ratio TS/T1 < 1depends only on N, (b) the normalized coefficient of variation (SD/T1)*SNR is a decreasing function of N, (c) example for SNR = 40 showing optimum saturation delay TS vs T1 for various N, and (d) for SNR = 40 showing SD vs T1 for various N.

Using brute force optimization, a fixed TS < T1 was found to be optimum or near optimum over a wide range of parameters. Using Eq 5, the CV = SD/T1 may be evaluated for the proposed sampling strategy of fixed TS. The ratio TS/T1 depends only on the number of measurements N (Figure 5(a)). For N = 1, the optimum saturation delay is approx. 84% of the T1 and decreases to 50% of T1 at N = 12.The normalized coefficient of variation (SD/T1)*SNR is a decreasing function of N (Figure 5(b)). For N = 12, the (SD/T1)*SNR is approx. 1.6; therefore, at SNR = 40 and T1 = 1000, the SD is calculated to be 40 ms. Plots for the optimum fixed TS and SD are shown for SNR = 40 and various N in Figure 5(c) and (d).

The fixed TS sampling was compared with brute force optimization which permitted recovery heartbeats. Results for calculation of CV for N = 9 and 12 at SNR = 40 for various strategies are listed in Table 1, for both 60 bpm and 120 bpm (in parentheses). For N = 12, T1 = 1200 ms, the fixed TS strategy is within 2% of a brute force optimized sampling which has a single measurement with recovery beats, for HRs 60 to 120 bpm. The brute force optimization chose 4 recovery heartbeats for 1 measurement, and 7 measurements at fixed TS (i.e., NS + [(0)1]^7^(4)1). For N = 9, T1 = 1200 ms, the fixed TS strategy without recovery beats is optimal (NS + [(0)1]^9^). For T1 = 500 ms and N = 12, the fixed TS strategy is within 13% of optimum at HR = 60 bpm and within 8% at a HR = 120 bpm. For T1 = 500 ms and for N = 9, the fixed TS strategy is within 7% and 1% at HRs 60 and 120 bpm, respectively. For T1 = 250 and N = 12 ms the fixed TS strategy is within 21% and 18% of the brute force optimization including recovery beats at HRs 60 and 120 bpm, respectively. Brute force optimization without recovery heart beats, choosing 9 measurements fixed at 150 ms and 3 measurements fixed at 800 ms (max), is within 6% of the best optimization which allows for recovery heart beats. For T1 = 250 ms and N = 9, the fixed TS strategy is within 12% and 11% of the brute force optimization allowing recovery beats at HRs 60 and 120 bpm, respectively. Brute force optimization without recovery heart beats, choosing 7 measurements fixed at 150 ms and 2 measurements fixed at 800 ms (max), is within 4.5% of the best optimization which allows for recovery heart beats.

**Table 1:** **Coefficient of variation (CV) for various sampling strategies for both N = 9 and N = 12 at SNR = 40, for HR = 60 bpm and 120 bpm (values in parentheses)**

**# SR measurements**		**N = 9**			**N = 12**	
T1 (ms)	250	500	1200	250	500	1200
NS + [(0)1]^N^ Uniform	0.0517 (0.0472)	0.0488 (0.0442)	0.0461 (0.0595)	0.0467 (0.0451)	0.0461 (0.0415)	0.0429 (0.0534)
NS + [(0)1]^N^ Fixed TS	0.0424 (0.0424)	0.0424 (0.0424)	0.0424 (0.0480)	0.0402 (0.0402)	0.0402 (0.0402)	0.0402 (0.0440)
NS + [(0)1]^3^ (1)1(2)1(3)1 SMART1Map	N/A	N/A	N/A	0.0670 (0.0512)	0.0564 (0.0566)	0.0596 (0.0563)
Brute force optimization without recovery beats	0.0394 (0.0394)	0.0424 (0.0424)	0.0424 (0.0424)	0.0353 (0.0353)	0.0402 (0.0402)	0.0402 (0.0402)
Brute force optimization allowing recovery beats	0.0377 (0.0383)	0.0396 (0.0419)	0.0424 (0.0424)	0.0333 (0. 0341)	0.0356 (0.0374)	0.0394 (0.0402)

### Optimization for unknown T1

**Figure 6:**
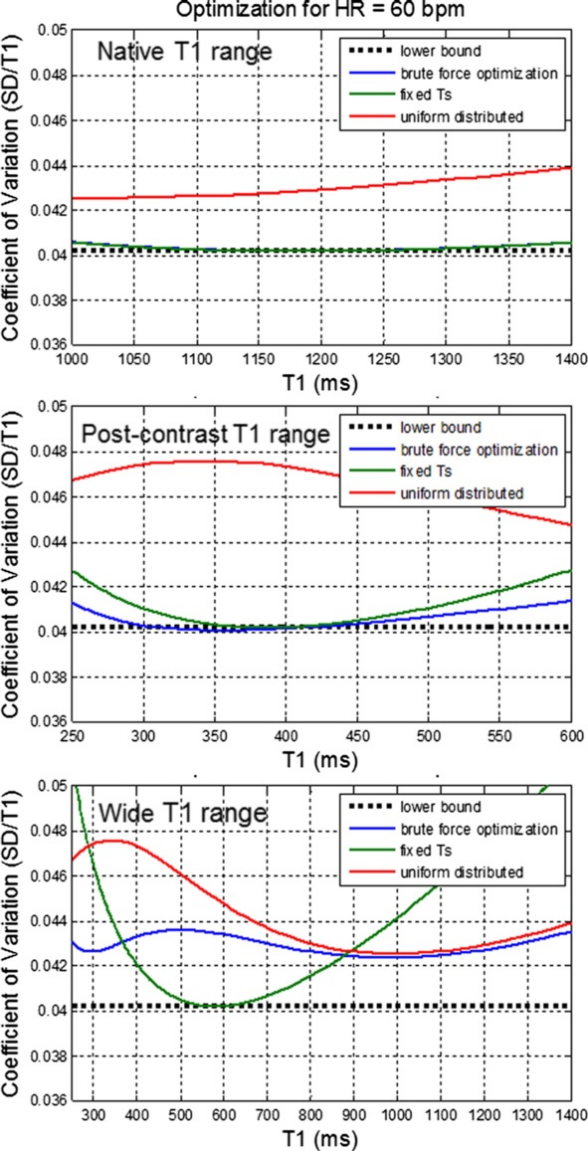
**Comparison of sampling strategies using NS + [(0)1]** ^12^ **with brute force optimization of the sampling scheme, uniform distribution, and fixed saturation delay against the lower bound (dotted) for 3 ranges of T1 uncertainty.**

Various sampling strategies were compared for the case of unknown T1 over 3 ranges of T1 corresponding to native T1 range, post-contrast T1, and a wide range spanning native and post-contrast. All protocols compared here were 13 heartbeat acquisitions with different sampling. The first set of plots (Figure 6) compare 3 strategies using NS + [(0)1]^12^ with different distribution of saturation delays: a brute force optimization without recovery heartbeats (blue), uniform distribution from 100 to 800 ms (red), and fixed TS (green). The fixed TS optimized for each range was 591, 193, and 290 ms for native, post-contrast, and wide ranges, respectively. The dotted black line is the optimum achievable SD, i.e., using fixed TS for each T1 which serves as a lower bound. Note that the fixed TS achieved approximately the same performance as the brute force optimization over the native contrast and post-contrast ranges, but deviates for the wide range. The saturation delays found by brute force search were (a) native range: NS + [2@550 ms, 10@600 ms], (b) post-contrast range: NS + [11@250 ms, 1@800 ms] (c) wide range: NS + [4@300 ms, 4@350 ms, 4@800 ms]. Brute force optimization over the native T1 range that included recovery beats chose a strategy of fixed TS sampling without recovery beats. Brute force optimization over the post-contrast T1 range that included recovery beats chose a strategy with a single recovery beat and was 12% better than the fixed TS strategy for this T1-range. Brute force optimization without recovery heart beats was within 8% of the brute force optimization which allowed recovery, but which increased the saturation delay for a single measurement (11 measurements at 250 ms and a single measurement at 800 ms).

**Figure 7:**
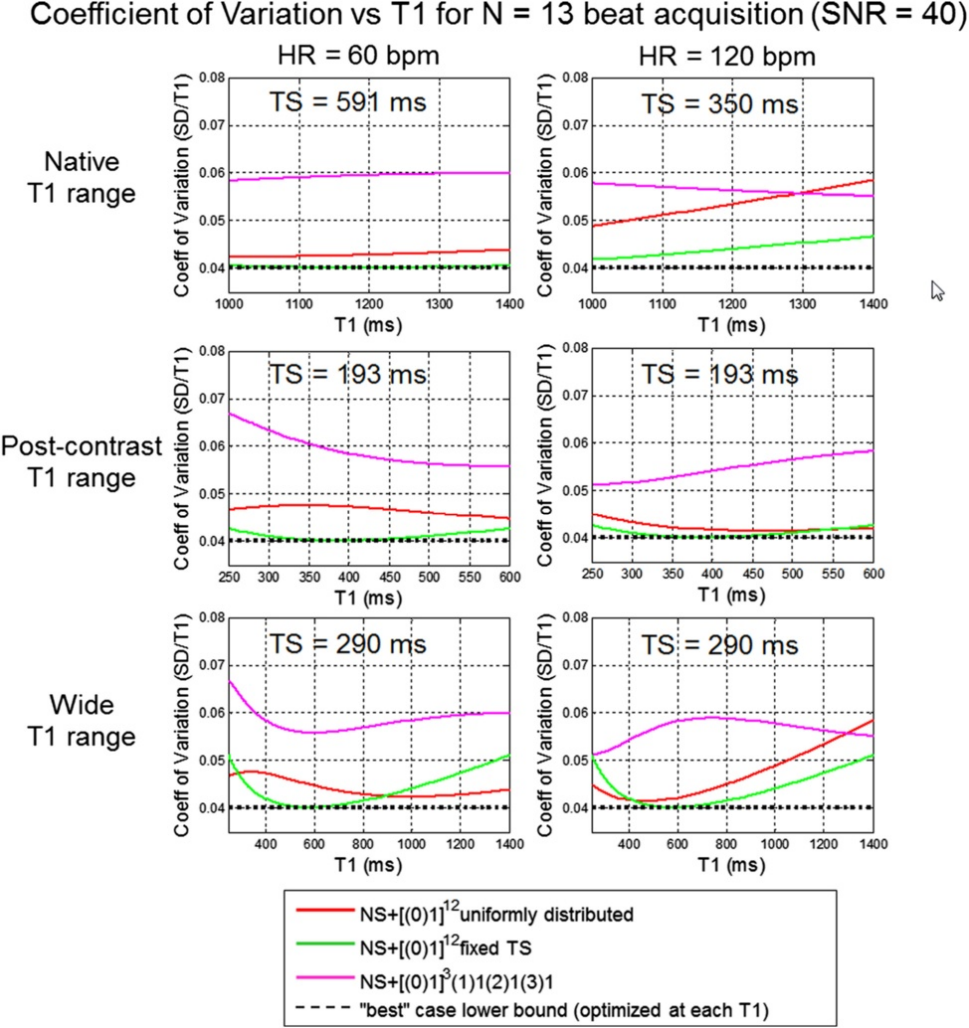
**Comparison of sampling strategies NS + [(0)1]** ^3^ **(1)1(2)1(3)1 and NS + [(0)1]** ^12^ **with uniform distribution and fixed saturation delay against the lower bound (dotted) for 3 ranges of T1 uncertainty and 2 heart rates.**

The second set of plots (Figure 7) compare the strategy NS + [(0)1]^3^(1)1(2)1(3)1 (magenta) with the NS + [(0)1]^12^ uniform distribution from 100 to 800 ms (red), and fixed TS (green) for heart rates (HR) of 60 and 120 bpm. The fixed TS optimized for each range are the same as Figure 6 with the exception of the native range at HR = 120 bpm for which 591 ms is not achievable; for this case a value of 350 ms is used. For native contrast and post-contrast ranges the fixed TS strategy has superior performance (lowest coefficient of variation across the range). The NS + [(0)1]^3^(1)1(2)1(3)1 strategy of recovery beats with uniform TS distribution, is significantly poorer than the fixed TS strategy.

### Phantom measurements

**Figure 8:**
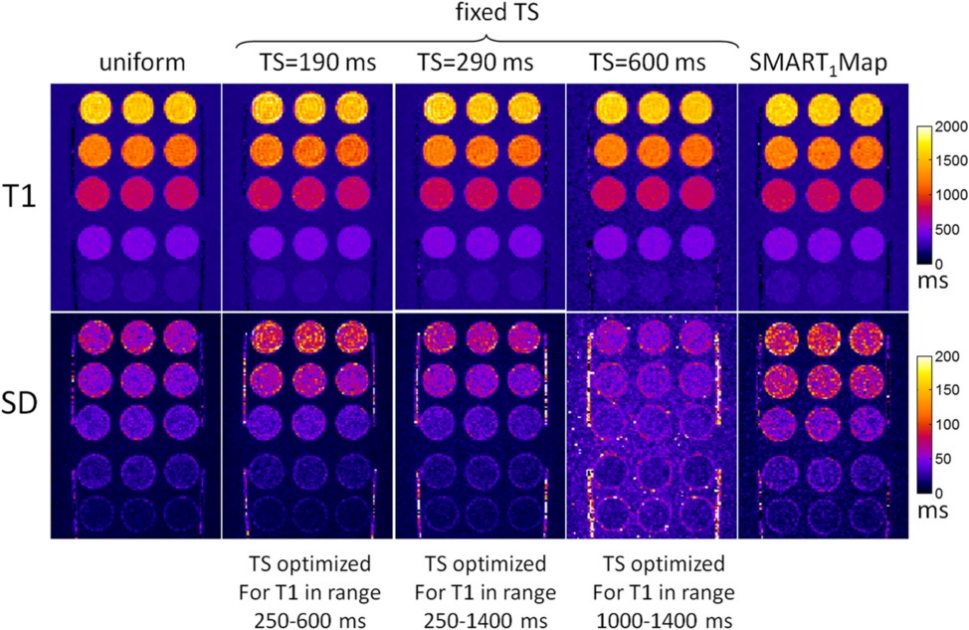
**Phantom T1 maps (top) and SD maps (bottom) for various protocols at HR = 60 bpm.**

**Figure 9:**
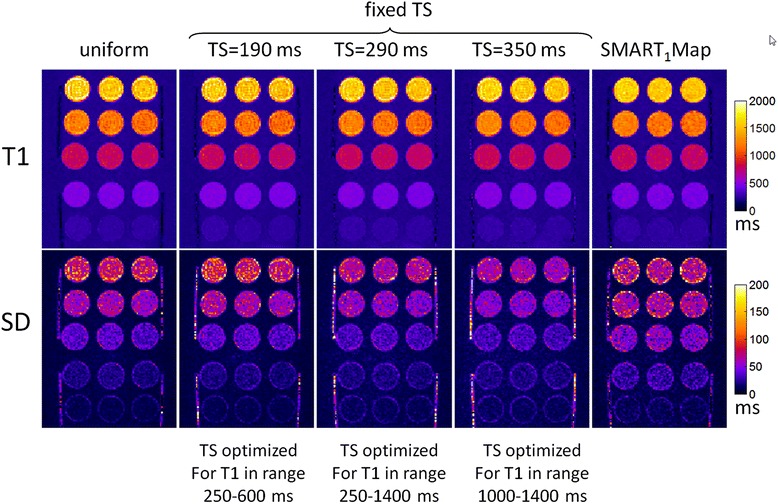
**Phantom T1 maps (top) and SD maps (bottom) for various protocols at HR = 120 bpm.**

**Figure 10:**
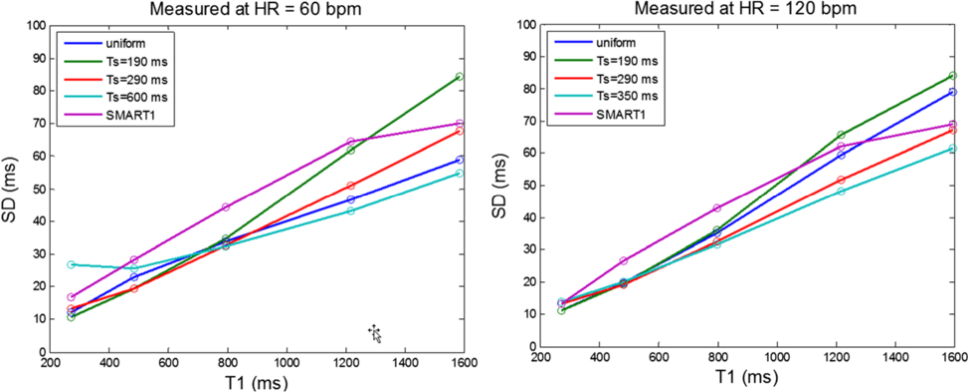
**Measured SD for phantom ROIs at HR = 60 bpm (left) and 120 bpm (right).**

**Figure 11:**
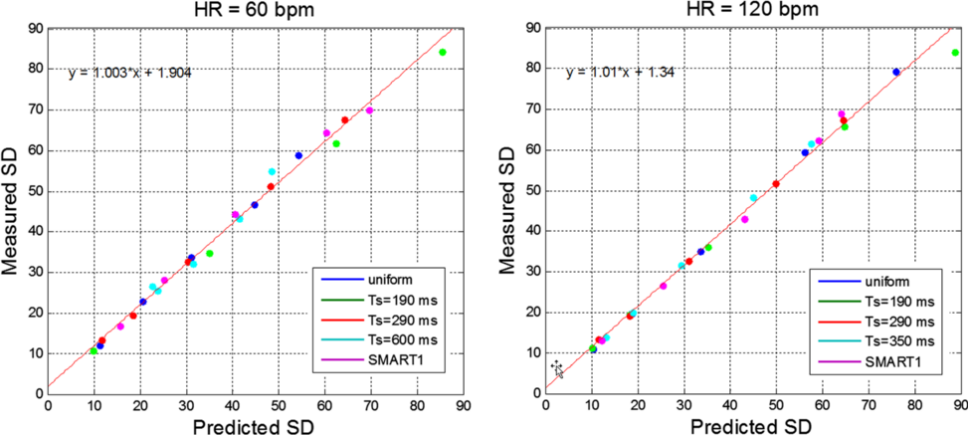
**Scatter plots of measured versus predicted SD for phantom ROIs at HR = 60 bpm (left) and 120 bpm (right).** Measurements plotted for 5T1-tubes with each of 5 protocols (Figure 10), with fit to all of the measured data (red line).

The sampling strategies compared in Figure 7 were evaluated using a T1-phantom. T1 and SD maps as well as SNR maps were reconstructed for each protocol and ROI measurements for 5 T1-tubes were compared with respect to measured SD and theoretical prediction as described in Figure 2. Images were acquired at both 60 and 120 bpm. The T1 of the agar gel tubes spanned the range of native myocardial T1 and for Gd contrast so fixed saturation delay protocols optimized for the 3 T1 ranges were used at each heart rate. The TS values were rounded slightly in comparison with the Figure 7 calculations. T1 and SD maps for 60 and 120 bpm are shown in Figures 8 and 9, respectively, and measured values of SD in 5 ROIs are graphed in Figure 10. The measured SD agreed well with the predicted SD for a given protocol, T1, and SNR (Figure 11). The SD values for the top rows in Figures 8 and 9 with longer T1 corresponding to native T1 range are smallest for the fixed TS protocol (TS = 600 ms for HR = 60 bpm and TS = 350 ms for HR = 120 bpm) as expected from Figure 7. The SD values for the lower rows with shorter T1 corresponding to post contrast range are smallest for the fixed TS protocol (TS = 190 ms) at HR = 60 and approx. the same as the uniform distribution for HR = 120 bpm, as expected from Figure 7. The NS + [(0)1]^3^(1)1(2)1(3)1 protocol performed significantly worse than the optimized fixed TS protocols over the native and post-contrast ranges for which they were respectively optimized.

### In-vivo measurements

**Figure 12:**
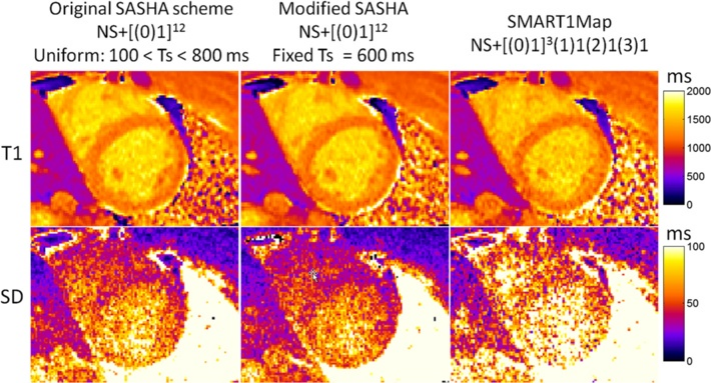
**In-vivo example of native T1 maps (top) and SD maps (bottom) using 3 SR-based T1 measurement protocols.**

**Figure 13:**
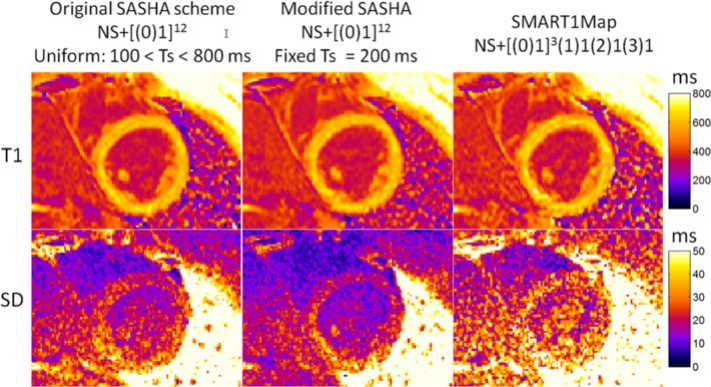
**In-vivo example of post-contrast T1 maps (top) and SD maps (bottom) using 3 SR-based T1 measurement protocols.**

**Figure 14:**
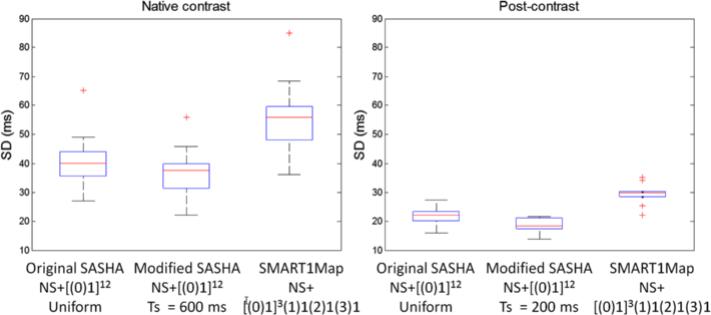
**Box and whisker plots of SD values (n = 10) in septal ROI comparing the 3 protocols for native contrast (left) and post-contrast (right).** Blue box represents interquartile range (IQR) with redline indicating the median.

T1-maps were acquired with 3 protocols on n = 10 subjects, both native T1 (pre-contrast) and post-contrast. Example T1 and SD maps are shown in Figures 12 and 13, for native and post-contrast examples, respectively. ROI measurements of the SD were made in the septum displayed as box and whisker plots (Figure 14). For pre-contrast measurements, the SD values for the SMART_1_Map approach are significantly higher than either the original SASHA protocol (p = 0.012) and the modified protocol (p = 0.0015); values for original and modified methods do not reach a statistically significant difference (p = 0.33). For post-contrast measurements, the SD values for the SMART1Map approach are significantly higher than either the original SASHA protocol (p < 0.001) and the modified protocol (p < 0.001); SD values for the modified protocol are less than the original protocol with statistical significance (p < 0.03) thereby demonstrating that the fixed TS protocols optimized for the specific T1-ranges have clearly improved precision.

## Discussion

The analytic formulation for parameter error (Eq 5) adds insight into finding optimal sampling strategies for saturation recovery T1 mapping. The first term in the denominator represents the uncertainty in an estimate of T1 given that the signal amplitude A is known and would be optimized at TS = T1 for all N. The 2 parameter fitting jointly estimates A and T1, and the second term in the denominator subtracts from the first term leading to an increase in SD and shifts the optimal TS < T1. Sampling with recovery heartbeats decreases the total number of measurements in order to maintain a fixed total duration for acquisition. The SD decreases as N increases (Figure 5(b)), therefore, recovery heartbeats can cause an increase in SD. However there is a point of diminishing returns where the added measurements do not significantly decrease the SD. At this point, the addition of a measurement with recovery beats can actually decrease the SD. While all of the measurements contribute to the joint estimation of T1 and signal amplitude, the short TS < T1 dominate the T1 estimation, and the NS anchor measurement dominates the amplitude estimate. At the point for which there are enough samples, the overall estimation benefits from a longer TS measurement (i.e., acquired at essentially full recovery after several recovery beats) to improve the amplitude estimate. The interplay between the 2 terms in the denominator is complex and depends N, T1, and the RR interval whereas the fixed TS sampling strategy is simple to implement.

In this study, we somewhat arbitrarily defined the native and post-contrast T1 ranges. In the case of native contrast, the range of actual myocardial T1 values are affected by disease conditions such as edema [16,17], iron deposition [18], and lipid deposition [19,20]. However, the optimal fixed saturation delay is fairly weakly dependent on the assumed native range 1000–1400 ms. In the case of post-contrast, the assumed 250–600 ms range is quite large. For a reduced target range for post-contrast T1 of 400–600 ms, the optimum fixed delays would increase to approximately 245 and 265 ms for NS + 12 and NS + 9 measurements, respectively. This further strengthens the conclusion that a fixed delay scheme is more optimal than other strategies.

In this study we optimized for the expected myocardial T1 and did not optimize for the blood signal. Although measurement of T1 in the blood is important for applications such as ECV, the blood signal is measured in a ROI and is generally at high SNR. Precision of the blood estimate is excellent and generally does not limit the ECV precision [11].

In the protocol comparison, the acquisition time was 13 beats for all 3 protocols to allow fair comparison, however shorter breath-holds may be desirable. In this case, the optimum saturation delay will slightly increase as shown in Figure 5. For instance for NS + 9 measurements, the optimum saturation delays increase slightly to approx. 210 ms and 640 ms for post-contrast and native T1 ranges, respectively. The total acquisition time can be moderately reduced without significantly decreasing T1 precision, with only an 8% increase in SD if the total duration is reduced to 9 beats from 13 (Figure 5(b)).

Three-parameter fitting of SASHA data had substantial increases in T1 variability at high heart rates [3,4]. However, these results show that 2-parameter SASHA maintains excellent precision with heart rates up to 120 bpm. The optimal fixed TS for native T1 values is achievable for within a single RR interval for heart rates of less than 80 bpm. At higher heart rates where the fixed TS time must be reduced, there is no significant reduction in precision, as demonstrated in simulations and phantom experiments.

This work demonstrates that brute force optimization of sampling including the possible addition of recovery heartbeats for SR T1 mapping does not improve precision as a function of total measurement time for the native T1 range. In the case of the post-contrast T1 range, there is an improvement in precision when increasing the delay of a couple of measurements. The SMART_1_Map strategy was previously shown to have less noise than the original SASHA for long T1 values at high HRs [10] when using 3 parameter fitting. In the current study, we have used SASHA 2 parameter fitting which has greatly improved precision compared with 3 parameter [3]. The optimum sampling strategy for 2 parameter fitting does not follow the same behavior as 3 parameter fitting and leads to a different conclusion. The 3-parameter fitting is much more sensitive to the estimation of amplitude terms.

SD maps may be used to compare and optimize protocols. SD maps also serve as a quality metric to assess the individual maps. SD values using the modified protocol have a tighter distribution whereas the SD estimates using the SMART_1_Map method are prone to outliers which are indicative of less reliable curve fitting.

The SASHA VFA with 2-parameter fitting has greatly improved performance over the prior SASHA with fixed excitation flip angle. Benefits of the VFA implementation are (1) a reduction of image artifacts which leads to an improved reproducibility, (2) improved SNR which benefits precision, and (3) reduction of the influence of readout which mitigates the loss in accuracy previously associated with 2-parameter fitting. With these benefits, the SASHA VFA 2-parameter method becomes highly attractive and competitive with inversion recovery protocols such as MOLLI [5]. From an absolute precision standpoint, the MOLLI protocols 5s(3s)3s and 4s(1s)3s(1s)2s used for native contrast and post-contrast acquisitions [3], respectively, are about 32% better than the optimized SASHA VFA with the same 11 heartbeat acquisition duration. However, the absolute accuracy of SASHA is superior to the MOLLI approach. More important than absolute accuracy is the reduced dependence of SASHA on variations in off-resonance and flip angle [3]. For native contrast, variations in off-resonance and flip angle can lead to artifactual variation in apparent T1 on the order of 5% or more when using MOLLI which is greater than the loss precision which is typically less than 2% on a pixel-wise basis. Respiratory motion correction of inversion recovery data is also more prone to errors than saturation recovery methods due to the large variation in image contrast.

## Conclusions

Sampling strategies for saturation recovery methods for myocardial T1-mapping have been optimized and validated experimentally. Improved precision may be achieved by using fixed saturation delays when considering native myocardium and post-contrast T1 ranges. The optimized TS was 591 ms, 193 ms, and 290 ms for native, post-contrast, and wide myocardial T1 ranges, respectively. Pixel-wise estimates of T1 mapping errors have been formulated and validated for SR fitting methods. The ability to quantify the measurement error has potential to determine the statistical significance of subtle abnormalities that arise due to diffuse disease processes involving fibrosis and/or edema and is useful both as a confidence metric for overall quality, and in optimization and comparison of imaging protocols.

## Abbreviations

MOLLI: Modified look-locker inversion recovery
SASHA: Saturation recovery single-shot acquisition
SMART_1_Map: Saturation method using adaptive recovery times for cardiac T1 mapping
SR: Saturation recovery
TS: Saturation time
ROI: Region-of-interest
SD: Standard deviation
CV: Coefficient of variation
NS: Non-saturated
